# Oral co-polymeric raft-forming nano gels for targeted empagliflozin delivery against stomach cancer (SGC7901)

**DOI:** 10.1016/j.heliyon.2024.e34074

**Published:** 2024-07-04

**Authors:** Nabil A. Alhakamy, Samaa Abdullah, Shadab Md, Akhalakur Rahman Ansari, Subrat Kumar Bhattamisra, Ibrahim M. Ibrahim, Hadil Alahdal, Abeer A. Altamimi, Rasheed A. Shaik

**Affiliations:** a Department of Pharmaceutics, Faculty of Pharmacy, King Abdulaziz University, Jeddah, 21589, Saudi Arabia; b Center of Excellence for Drug Research Pharmaceutical Industries, King Abdulaziz University, Jeddah, Saudi Arabia; c Mohamed Saeed Tamer Chair for Pharmaceutical Industries, Faculty of Pharmacy, King Abdulaziz University, Jeddah, Saudi Arabia; d Natural and Health Sciences Research Centre, Princess Nourah bint Abdulrahman University, P.O. Box 84428, Riyadh 11671, Saudi Arabia; e Center of Nanotechnology, King Abdulaziz University, Jeddah 21589, Saudi Arabia; f Department of Pharmaceutical Technology, School of Medical Science, Adamas University, Kolkata, India; g Department of Clinical Pharmacology, Faculty of Medicine, King Abdulaziz University, Jeddah 21589, Saudi Arabia; h Department of Biology, Faculty of Science, Princess Nourah bint Abdulrahman University, Riyadh, 84428, Saudi Arabia; i Department of Pharmacology and Toxicology, Faculty of Pharmacy, King Abdulaziz University, Jeddah, Saudi Arabia

**Keywords:** Raft, Gastro-retentive, Controlled release, Stomach cancer, Polymeric nanoparticles, Empagliflozin

## Abstract

Empagliflozin (EMP) is known for its poor safety and efficacy profile due to its fast body distribution and poor solubility. Accordingly, an oral long-acting and floating/raft-forming nano gel was optimized to release coated EMP nanoparticles, and the released EMP nanoparticles showed enhanced dissolution compared to raw EMP particles. To repurpose EMP for cancer treatment, EMP shows anti-cancer and anti-inflammatory effects against cancer cells. EMP nanoparticles were characterized using FT-IR, PXRD, SEM, EMP encapsulation assay, and release studies. The raft-forming gel encapsulating the EMP was optimized and characterized. The EMP co-polymeric nanoparticles were studied to investigate EMP anti-cancer and anti-inflammatory activities against stomach cancer cells. The solubility of EMP nanoparticles was enhanced in 0.1 N HCl and pH 6.8 by 5 and 12 folds, respectively, compared to raw EMP powder. The particle size and zeta-potential values of improved EMP nanoparticles were 135.40 ± 18.60 nm, and −19.30 ± 0.80 mV, respectively. FT-IR, PXRD, SEM and TEM characterizations revealed polymeric coating of EMP particles. The study suggested that this optimized controlled-release raft-forming gel is a promising local oral approach against stomach cancer. The repurposing of EMP co-polymeric nanoparticles for stomach cancer and associated gastritis treatment was justified.

## 1 Introduction

Stomach cancer is a major public health concern around the world. The incidence and frequency of stomach cancer are increasing at an alarming rate. As a result, there is an unmet need for novel therapeutic development or repurposing of existing therapies to effectively control and treat stomach cancer [1]. Empagliflozin (EMP) is a newer type of diabetes medication that works by inhibiting the proximal tubular sodium-glucose co-transporter-2 (SGLT-2) and is hence referred to as an SGLT-2 inhibitor [2]. Previously published studies have shown the potent anti-cancer potential of EMP against cervical cancer via modulation of redox status and AMPK pathways. Another recently published study showed the anti-cancer potential of EMP and metformin via modulation of NF-kB/TNF-α, MMPs, Akt, Ki-67, and VEGF axis [3]. EMP was also reported to have potent anti-inflammatory potential, and thus, this drug is a promising candidate to address stomach cancer. In addition, the development of an EMP controlled-release gastro-retentive system could enhance its poor safety and efficacy profile which arises due to the fast body distribution and poor solubility [4]. As a result, the goal of the current study was to design and produce EMP-loaded Alginate (SA)-Ploy Ethylene Glycol (PEG) nanoparticles (EMP-ALG-PEG-NPs) to treat stomach cancer. To that end, an oral long-acting and gastro-retentive raft-forming nano gel with EMP-ALG-PEG-NPs was optimized and loaded. The SA is a hydrophilic and linear polysaccharide composed of d-mannuronic acid and l-guluronic acid [5]. The SA, of a low molecular weight grade (5000–20,000 Da), comprises multiple hydroxyls and carboxylate groups. Alginate, a biodegradable polymer, has been extensively studied as a safe and effective carrier for anti-cancer drugs for different cancers. Alginate polymer offers a non-toxic, stable carrier system with significant encapsulating properties [5]. PEGs, in comparison, are hydrophilic synthetic oligomers or polymers derived from ethylene glycols. Their chemical formula is H-(O–CH2–CH2)_m_-OH, where m is the degree of polymerization, and they contain both hydrophobic and hydrophilic ethylene units (CH_2_–CH_2_). The PEG (6000 g/mol) polymer is distinguished by the poly ether groups and its ability to act as a surfactant in dosage forms. Therefore, SA and PEG polymers could bind with the hydroxyl groups of EMP after coating and decrease the EMP's aromatic rings' interfacial tension through hydrogen bond formation with SA and PEG [[6], [7], [8]]. Subsequently, these attempts were combined with the formation of the NPs to increase stability and biological activity sensitivity [[9], [10], [11], [12], [13], [14], [15]].

Nanoparticles (NPs) are nanosized drug delivery systems ranging from 10 to 1000 nm. Because of their reduced size and enhanced sensitivity and stability, it has been extensively used for targeting drugs against different types of cancer [16]. In one study, alginate NPs with a size range of 85–300 nm loaded with prednisolone were used for targeted colonic delivery [17].

EMP is known for its poor safety and efficacy profile due to its fast body distribution and poor solubility. An oral long-acting and floating/raft-forming nano gel was optimized to release coated EMP nanoparticles, and the released EMP nanoparticles showed enhanced dissolution compared to raw EMP particles. In this study, the EMP-ALG-PEG-NPs were developed and characterized using FT-IR, PXRD, SEM, EMP encapsulation assay, EMP solubility studies, and release studies compared to the EMP-free drug. In addition, the controlled release raft-forming gel encapsulating the EMP was optimized, developed, and characterized. The EMP co-polymeric NPs were studied to investigate EMP anti-cancer, anti-inflammatory effects on stomach cancer cells, and dissolution enhancement effects compared to free EMP.

## 2 Materials and methods

### 2.1 Materials

Jamjoom Pharmaceuticals, Jeddah, Saudi Arabia, provided empagliflozin as a free sample. Sigma-Aldrich, USA, and TCI America, USA, provided sodium alginate (low molecular weight grade; 5000–20,000 Da) and polyethene glycol (6000 g/mol, respectively. ATCC (Manassas, VA, USA) provided the stomach cancer cell line (SGC7901). Gibco, London, UK provided Dulbecco's Modified Eagle Medium (DMEM) supplemented with 10 % fetal bovine serum (FBS), penicillin, and streptomycin.

### 2.2 Preparation and optimization of EMP-ALG-PEG-NPs

In water, a stock solution of 30 mg/mL SA-low molecular weight grade (5000–20,000 Da, CAS. NO. 9005-32-7) was prepared. Afterwards, 100 mg of EMP was added using water to different amounts of PEG (6000 g/mol, CAS. NO. 25322-68-3) to complete 4.5 mL dispersion using a magnetic stirrer (Table 1). Following that, ultrasound nanomaterial dispersion equipment with a high shear probe sonicator (20 kHz, 2000 W, and 20 mm probe diameter, Biosafer ultrasonicator, China) was used for 15 min to add various colloidal dispersions of EMP and PEG to a 3.5 mL of SA stock solution [12,13].

**Table 1:** Illustration of the different EMP^#^ formulations' components for optimization.

Formula No.	PEG concentration (mg/mL)	SA equivalent amount (mg/mL)	PEG: SA ratio ^@^	Total volume (mL)	Particle Size (nm)^a^	PDI^a^
1	3.75	13.13	1:4	8	635.10 ± 18.90	0.71 ± 0.03
2	7.5		1:2		545.40 ± 20.90	0.78 ± 0.03
3	11.25		1:1		135.40 ± 18.60	0.44 ± 0.13

a For n = 3 ± SD; @ Approximate ratio; # EMP concentration is 12.5 mg/mL.

The particle size of the various liquid combinations was determined using a zeta-sizer (Malvern Zeta-sizer Nano ZS, Malvern Instruments Ltd., UK). The zeta potential value of the best EMP-ALG-PEG-NPs system with the smallest particle size and homogeneous PDI was also investigated. To extract the drug NPs from the polymeric system, 2 mL of the optimized co-polymeric system was diluted with 2 mL of distilled water and vortexed for 30 s. The mixture was then centrifuged for 15 min at 3000 rpm to allow the larger particles to settle, and samples for zeta size measurement were obtained from the supernatant layer [15]. Furthermore, drug NPs encapsulation effectiveness was assessed using UV-scanning (Shimadzu, Japan) of the collected samples at 272 nm using the formula shown below [7,11].

EMP−ALG−PEG−NPsencapsulationefficiency=(SupernatantEMPamount)/(TotalEMPamount)×100

### 2.3 Characterizations of optimum EMP-ALG-PEG-NPs

#### 2.3.1 Fourier transform-infrared (FT-IR)

FT-IR spectrum analysis was performed on samples of EMP, PEG, SA, physical mixture, blank co-polymeric NPs, and EMP-ALG-PEG-NPs (10 mg) (Thermo-Scientific, Nicolet iS10, USA). The samples were scanned at 500 to 4000 cm^−1^ resolution [18].

#### 2.3.2 Powder X-ray diffractometer (PXRD)

EMP, PEG, SA, physical mixture, blank co-polymeric NPs, and EMP-ALG-PEG-NPs were studied using a Maxima XRD-7000X powder X-ray diffraction (PXRD) system (Rigaku, Ultima IV XRD, Japan). During the procedure, X-rays were generated at 40 kV and 40 mA using nickel-filtered Cu-Kb reduction. The scan range (2θ) was 5–70° at a speed of 10° per minute [14].

#### 2.3.3 Morphology and dispersion investigations

Scanning electron microscopy (SEM) was used to examine the structure and particle distribution of EMP, PEG, SA, physical mixture, blank co-polymeric NPs, and EMP-ALG-PEG-NPs in powder form (JSM-7600F, Jeol, Japan). A 30 kV voltage was used to examine the dried material. Moreover, Transmission Electron Microscopy (TEM) was used to investigate the morphology and size of EMP-ALG-PEG-NPs (JEM-F200, Jeol, Japan). TEM photos were acquired after negative staining (phosphotungstic acid, 2 %) was performed by depositing one drop of the material, after appropriate dilution, over a copper grid [14].

### 2.4 Solubility assessment

Centrifuge tubes of 15 mL were filled with samples comprising more than 10 mL of the fluid under inquiry. The materials were vortexed for 15 min before being placed in a shaking water bath for 72 h. After centrifuging the material, the supernatant was dissolved in a precise amount of ethanol. The EMP content was calculated using UV-spectrophotometry at 272 nm [19].

### 2.5 Formulation and optimization of raft-forming gel encapsulating EMP-ALG-PEG-NPs

Optimum EMP-ALG-PEG-NPs using a 12.5 mg/mL EMP concentration was suspended in different volumes of SA-high molecular weight (600,000 g/mol) of 3 % stock solution as mentioned in Table 2 using a stirrer machine for 10 min [[20], [21], [22]].

**Table 2:** Raft-forming gels components of EMP-ALG-PEG-NPs.

#	SA^a^-gel (mg/ml)	PEG: SA** (mg/mL)	EMP (mg/mL)
F1	6	11.25 : 13.13	12.5
F2	12		
F3	18		

a High molecular weight; ** Low molecular weight.

### 2.6 EMP release analysis

EMP release was examined in three raft-forming gels (F1–F3), the EMP raw material, and the optimum EMP-loaded co-polymeric NPs at 37.00 ± 0.05 °C in a shaker at 75.00 ± 0.05 rpm. The EMP raw material was dispersed in distilled water until a 12.5 mg/mL EMP concentration which was similar to the EMP-ALG-PEG-NPs [3,23]. The groups were placed in dialysis bags with a cut-off value of 12,000–14,000 (Sigma, USA) that were submerged in 300 mL of the release media. For the first 2 h, the groups were immersed in 0.1 N HCl, a simulated stomach fluid with a pH of 1.2. The groups were then immersed in a phosphate buffer with a pH of 6.8, which resembled proximal intestine fluid. To imitate sink body conditions, the medium was completely replaced after 1 h [20].

### 2.7 Raft characterization analysis

The raft-forming gels in Table 2 were characterized by measuring raft strength, volume, and resilience. Raft characterizations and release were used as selection criteria for the optimum raft forming gel encapsulating the EMP-loaded co-polymeric NPs [24,25]. The volume, weight, strength, and resilience of the raft produced in 0.1 N HCl were measured.

#### 2.7.1 Raft density

In a pre-weighed 250 mL glass beaker (W1), a volume (15 mL) was added to 150 mL HCl. The position of the raft's top was marked on the outside of the beaker after 30 min of raft construction. The total weight of the beaker and contents (W2) was obtained after raft creation. The raft was carefully removed from the beaker after gently decanting off the supernatant liquid and placing it into a pre-tared watch glass. The supernatant liquid was drained after 30 s, and the raft was weighed (W3). The leftover liquid was scraped with a paper towel from the interior of the beaker before it was refilled with water to the specified position and weighed (W4). The volume (mL) of each raft was calculated using the weights (gm) obtained as (W4–W1)-(W2–W1–W3), assuming a subnatant density of 1.

#### 2.7.2 Raft durability

Each formula (15 mL) was mixed with 150 mL of 0.1 N HCl and stored at 37 °C in a 50 mL centrifuge tube. After 30 min, the tube was sealed and swirled at 20 rpm in a roller mixer to imitate stomach agitation (Roller Mixer-205 RM, Hawashin Tech. Company, Korea). The raft was visually evaluated every 2, 5, 10, 20, 30, 45, and 120 min, or until it sedimented or was no longer visible. Raft durability was calculated using the period after which the raft could no longer be seen.

#### 2.7.3 Raft strength

Rafts were created in a 250 mL glass beaker over 30 min by adding a formulation volume (15 mL) to 150 mL of 37 °C 0.1 N HCl. The rafts were removed, and the force (N= Kg.m/s^2^) needed by a stainless-steel cone to break the raft was measured using a Texture Analyzer XT Plus C (Stable Micro Systems, UK). The strength was expressed in terms of mass (g) after dividing the force required by the constant acceleration of the stainless-steel cone.

### 2.8 Cell viability assay

To evaluate the therapeutic efficacy of developed NPs after the release from the raft, anti-cancer activity was determined against the stomach cancer cell line, *i.e.,* SGC7901, via MTT assay. For this purpose, SGC7901 cells were first incubated in a 96-well plate at a temperature of 37 °C for 24 h. This study was carried out with a cell density of 5 × 10^3^ SGC7901 cells/well in a humidified CO_2_ chamber. Then various selected treatment groups of SGC7901 such as ALG-PEG-NPs, EMP-ALG-PEG-NPs, and EMP in comparison to the control were evaluated for cell viability after retaining for 24 h. The positive control was the untreated cancer cells, and the negative control was the culture media without the cells. All collected samples from cell plates were centrifuged and the collected supernatant (100 μl) was replaced with DMSO. Then, these dilutions were placed in a CO_2_ incubator at 37 °C for 4 h. After incubation, samples were analyzed by taking absorbance through a microplate at 570 nm in triplicate [2,26,27].

### 2.9 ELISA test

For the level of IL-6 and TNF-α, commercially available ELISA kits (Abcam, UK and Invitrogen, USA, respectively) were used, and inflammatory and apoptotic markers were estimated as per the manufacturer's instruction [2,26,27].

### 2.10 Statistical interpretation

Statistical analysis was performed using one-way ANOVA followed by Tukey's Multiple Comparison Test, and data were represented in terms of mean±standard deviations (SD). p ≤ 0.05 was regarded as statistically significant in the statistical analysis, which was performed using Graph Pad Prism 4.0 software (Graph Pad Software San Diego, USA).

## 3 Results and discussion

### 3.1 Selection and optimization of EMP-ALG-PEG-NPs

In water, a stock solution of 30 mg/mL SA-low molecular weight grade (5000–20,000 Da, CAS. NO. 9005-32-7) was prepared. Afterwards, 100 mg of EMP was added using water to different amounts of PEG (6000 g/mol, CAS. NO. 25322-68-3) to complete 4.5 mL dispersion using a magnetic stirrer (Table 1). Following that, ultrasound nanomaterial dispersion equipment with a high shear probe sonicator (20 kHz, 2000 W, and 20 mm probe diameter, Biosafer ultrasonicator, China) was used for 15 min to add various colloidal dispersions of EMP and PEG to 3.5 mL of SA stock solution [12,13]. According to Table 1, formulation no. 3 showed the smallest and the most homogenous EMP-ALG-PEG-NPs compared to the other formulations. Particle size analysis of the optimum EMP-ALG-PEG-NPs system (Formula 3) showed a particle size average of 135.40 ± 18.60 nm. The zeta potential and PDI values of formula 3 were −19.30 ± 0.80 mV and 0.44 ± 0.13, respectively, which confirmed the medium aggregation tendency, homogeneity and SA coating of the generated EMP-ALG-PEG-NPs [28]. As a result, the EMP NP encapsulation efficiency of the optimum co-polymeric system was 89.33 ± 0.23 %. In sum, the difference between the three different formulations was the PEG: SA ratio; and the highest ratio was in formulation no. 3. This difference could lead to optimum shearing and coating of the EMP, in which the PEG was a surfactant that decreased the interfacial tension between the EMP and SA [6,8,14,29]. Interestingly, the PEG, SA, and EMP amounts were 11.25, 13.13, and 12.5 mg/mL in formulation no. 3, respectively.

### 3.2 Characterizations of optimum EMP-ALG-PEG-NPs

#### 3.2.1 Fourier transform-infrared (FT-IR)

**Fig. 1:**
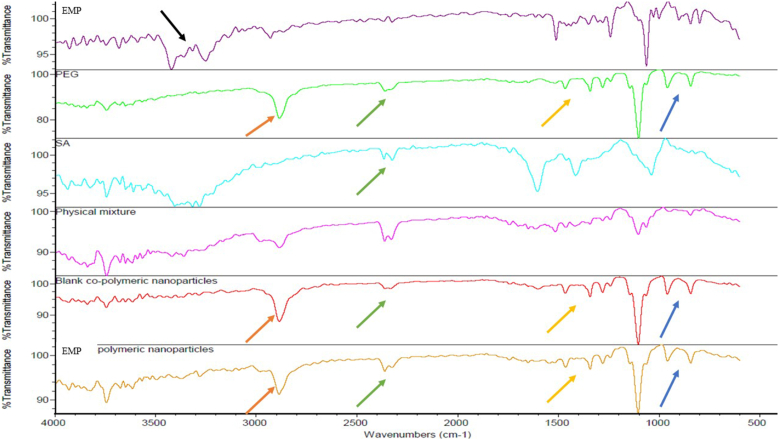
The FT-IR spectra for the EMP, PEG, SA, physical mixture, blank co-polymeric NPs, and EMP co-polymeric NPs.

As shown in Fig. 1, the EMP spectrum shows consistency with the literature regarding the presence of absorption bands at 3435 cm^−1^ (O–H stretching), 3260 and 3000 cm^−1^ (aromatic C–H stretching), 2900 and 2800 cm^−1^ (aliphatic C–H stretching), and 1050 cm^−1^ (C–O stretching) [30]. Meanwhile, the PEG spectrum shows the ether bond (C–O) stretching at 3600–3400 cm^−1^ and the aliphatic ethylene (C–H) or (C–C) bands at 1100–1000 cm^−1 6^. The peaks of interest and similarity between PEG and the composite materials, i.e., 2800–2900 cm^−1^, were added to Fig. 1 as an indication of the dominant PEG coating of the EMP nanoparticles [31]. In addition, the SA spectrum is distinguished by the carboxylate (C–O) and (H–O) stretching at 4000–3500 cm^−1^ and the aromatic (C–C) and (H–C) bands between 1600–1000 cm^−1 10^. As a comparison, the physical mixture spectrum shows blends of EMP, PEG, and SA peaks. However, the blank co-polymeric NPs spectrum confirms the outer functionalities of the system, in which the spectrum is divided into SA's carboxylate (C–O) and (H–O) stretching between 4000–3500 cm^−1^, and PEG had a similar spectrum between 3500 and 500 cm^−1^ for ether bond (C–O) stretching, 2800–2900 cm^−1^, and the aliphatic ethylene (C–H) or (C–C) bands. Meanwhile, the EMP-ALG-PEG-NPs spectrum has a similar spectrum to the blank one, but with a higher band intensity between 4000–3500 cm^−1^ of SA and PEG's carboxylate (C–O) and (H–O) stretching. The previous intensification of the 4000–3500 cm^−1^ band could be due to EMP hydrogen bond formation [10]. Interestingly, EMP could be coated with SA and PEG to enhance dissolution. Therefore, the SA and PEG polymers could bind with the hydroxyl groups of EMP after coating and decrease the EMP's aromatic rings' interfacial tension through hydrogen bond formation with SA and PEG. This type and order of binding could encapsulate the hydrophobic functionalities in the EMP inside the particles to expose the hydroxyl groups with the PEG and SA coating [14,30]. The main and related peaks are marked with the similar colors in Fig. 1 between the groups.

#### 3.2.2 Powder X-ray diffractometer (PXRD)

**Fig. 2:**
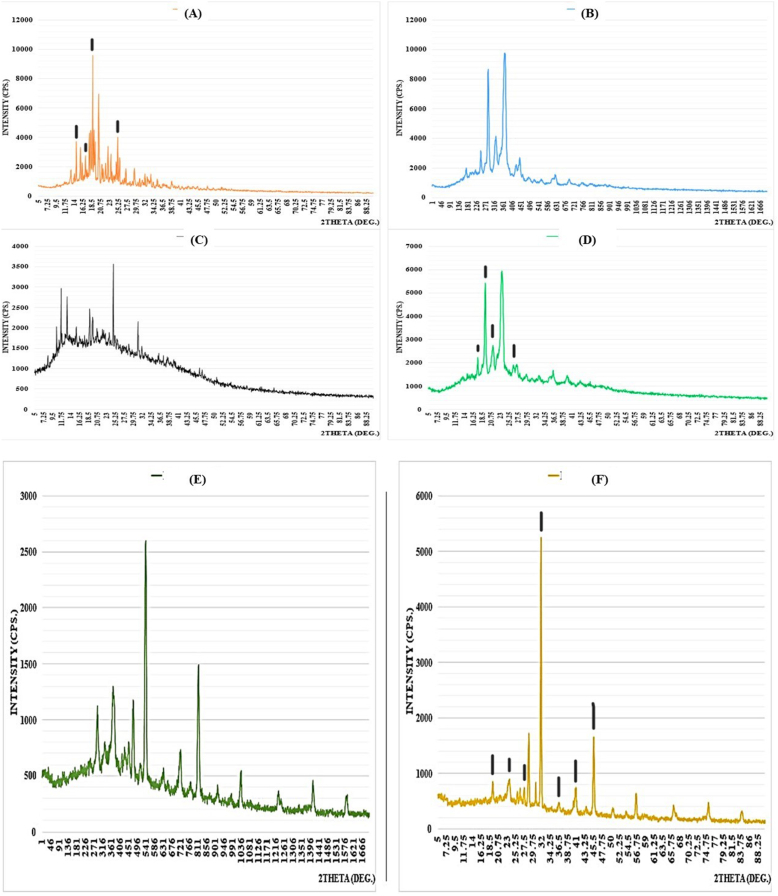
The PXRD diffractograms for the (A) EMP, (B) PEG, (C) SA, (D) physical mixture, blank co-polymeric NPs, and (E) EMP co-polymeric NPs.

As shown in Fig. 2A and B, EMP and PEG have crystalline diffractograms compared to that of SA. The physical mixture shows a combination of both SA and PEG diffractograms (Fig. 2C and D). In comparison, blank NPs have a diffractogram of different characters compared to that of crystalline PEG, which could be due to the higher SA amounts and increased influence on NPs compared to PEG. Interestingly, the EMP-ALG-PEG-NPs have a less noisy diffractogram than the blank one due to the EMP crystals. The previous result confirms the crystalline nature of EMP-ALG-PEG-NPs, which might add to their stability (Fig. 2E and F) [[32], [33], [34]].

The diffractograms of the EMP, physical mixture and EMP copolymeric nanoparticles were analyzed in Table 3 based on the theta (θ, degree) of marked peaks. The analysis included the use of Bragg's Law to determine the inter-planner spacing (d*hkl*, A), Miller indices (h, k, l) and lattice constant (a, A) assuming the cubic structure as the equation below [35].

2*dhkl*sin(θ)=n*λ

$$\sqrt{\frac{dhkl}{\left(h^{2}+k^{2}+l^{2}\right)}}=a$$

**Table 3:** The lattice constant (a) calculation using Inter-planner spacing (d_hkl_), peak values (θ) and miller indices (h, k, l).

	θ (degree)	λ (A)	n	d_hkl_ (A)	h	k	l	a = b = c (cubic) (A)
**EMP**	14.00	1.54	1	0.78	3.00	6.00	6.00	0.10
	16.25			−1.49	–	–	–	–
	18.50			−2.25	–	–	–	–
	25.25			6.58	1.00	1.00	1.00	1.48
**Physical mixture**	16.25	1.54	1	−1.49	–	–	–	–
	18.50			−2.25	–	–	–	–
	20.50			0.77	3.00	6.00	6.00	0.10
	25.25			6.58	1.00	1.00	1.00	0.29
**EMP-ALG-PEG-NPs**	18.50	1.54	1	−2.25	–	–	–	–
	23.00			−0.91	–	–	–	–
	27.50			1.10	5.00	1.00	1.00	0.12
	32.00			1.40	4.00	0.00	0.00	0.13
	36.50			−0.83	–	–	–	–
	41.00			−4.85	–	–	–	–
	45.50			0.77	3.00	6.00	6.00	0.10

The inter-planner spacing (d_hkl_, A) values was calculated using the first equation (Bragg's Equation), mentioned θ values mentioned in Table 3, Diffraction peak order (n) as it equals to 1, and X-ray wavelength (λ) to be 1.54 A. Using the International Center Diffraction Data (ICDD) card and based on the d_hkl_ found, the values of the miller indices (h, k, l) were found to be used for the lattice constant (a) calculation in the different groups. In Table 3, the EMP and physical mixture crystal and lattice parameters were similar. The EMP's crystals peaks in the physical mixture diffractogram were with lower intensities due to the SA and PEG dilution effect. However, the EMP copolymeric nanoparticles' crystal lattice parameters were different than the other groups indicating the effect of the SA and PEG coating on the EMP particles. This might influence the dissolution enhancement of EMP using EMP copolymeric nanoparticles [35].

#### 3.2.3 Morphology and dispersion investigations

**Fig. 3:**
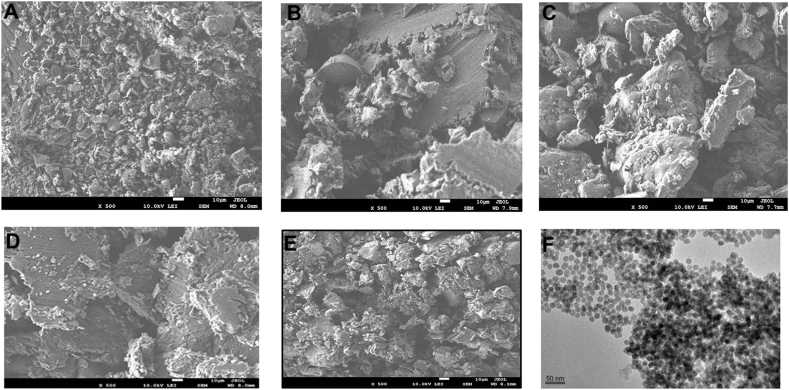
The SEM images for the EMP (A), PEG (B), SA (C), blank co-polymeric NPs (D), and EMP-ALG-PEG-NPs (E). The TEM images for EMP-ALG-PEG-NPs (F).

As shown in Fig. 3A–C for SEM imaging, the EMP crystalline particles are smaller than the PEG crystalline and SA amorphous particles. However, the blank co-polymeric matrix of SA and PEG using their optimized amounts are different in morphology than the original raw SA and PEG due to the film formation ability of SA after hydration and drying (Fig. 3D) [11,31]. In comparison, the optimum EMP-loaded co-polymeric matrix crystals are smaller than the EMP, as marked in Fig. 3E. After all, the optimum EMP-loaded co-polymeric matrix is comprised of regular shapes compared to the EMP crystals. Moreover, EMP deposition and encapsulation on the flakes of the blank co-polymeric matrix were observed in the optimum EMP-loaded co-polymeric matrix. The optimum EMP-nanoparticles TEM image (Fig. 3F) was found to have spherical nanoparticles with average size of monomeric nanoparticles of less than 50 nm, but the image shows tendency of aggregation that could be correlated to the particle size analysis results.

### 3.3 Solubility assessment

In Table 4, the solubility of EMP nanoparticles was enhanced in the 0.1 N HCl and pH 6.8 by 5 and 12 fold, respectively, compared to raw EMP powder. After all, the EMP co-polymeric NPs' had more enhanced solubility in the simulated intestinal media (pH 6.8) than the simulated gastric media (pH 1.2) compared to the raw material, which might be due to SA coating of the NPs [4,5,10]. In addition, the SA and PEG polymers bonded with the hydroxyl groups of EMP after coating through hydrogen bond formation to decrease the EMP's aromatic rings' interfacial tension. This type and order of binding could encapsulate the hydrophobic functionalities in the EMP inside the particles to expose the hydroxyl groups with the PEG and SA coating as suggested in the FT-IR results [32,36].

**Table 4:** Free EMP and EMP-ALG-PEG-NPs’ solubilities in 0.1 N HCl and pH 6.8 media.

Group	EMP Solubility in 0.1 N HCl (mg/mL)^a^	EMP Solubility in pH 6.8 (mg/mL)^a^
**Free EMP**	0.28 ± 0.01	0.14 ± 0.02
**Co-polymeric NPs**	1.37 ± 0.05	1.56 ± 0.04

a For n = 3 ± SD.

### 3.4 EMP release analysis and raft characterizations

**Fig. 4:**
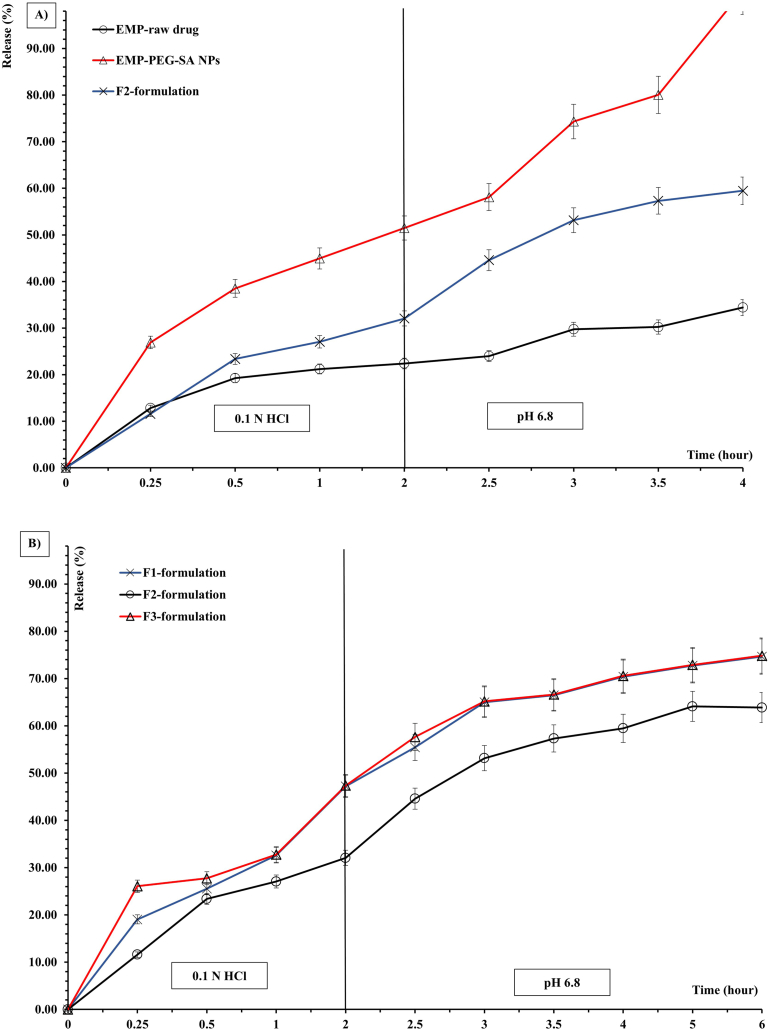
The release analysis for the (A) EMP raw material, the EMP-loaded co-polymeric NPs, and (B) the different formulations.

The coated EMP NP's release rate was faster than free EMP by more than two fold in the simulated gastric media (Fig. 4A). In addition, the coated EMP-ALG-PEG-NPs release rate in the simulated intestinal media was more than three fold higher than the free EMP (Fig. 4A). As a result, the dissolution enhancement of EMP using the SA and PEG coating and hydrogen bond formation was confirmed in the simulated gastric media and simulated intestinal media, which could be applied after release from the formation of the optimized raft. The SA and PEG polymers bonded with the hydroxyl groups of EMP after coating and decreased the EMP's aromatic rings' interfacial tension through hydrogen bond formation with SA and PEG [13].

For the comparison between the different raft-forming gels containing the EMP-ALG-PEG-NPs, the release analysis of the F1, F2 and F3 containing 6, 12, and 18 mg/mL, respectively, of high molecular weight SA (Table 5) were studied using Initial Dissolution Rate (IDR) and Mean Dissolution Time (MDT) for the first 2 h (MDT_1.2_) and last (MDT_6.8_) 4 h [9]. The IDR is the dissolved drug amount (mg) over the first 30 min per min. MDT was calculated using the following equation;

$$MDT=\sum_{i=1}^{n}t\;mid*\Delta M/\sum_{i=1}^{n}\Delta M$$

**Fig. 5:**
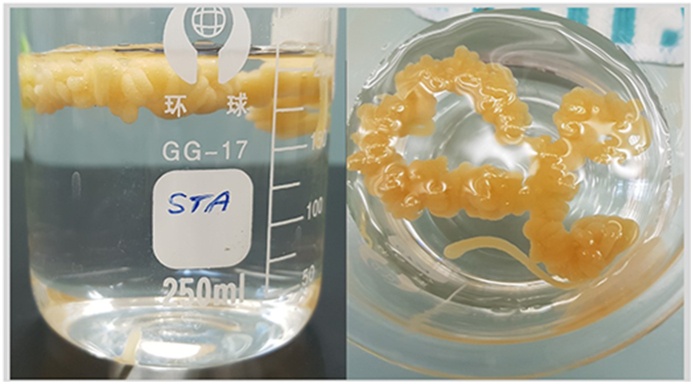
The raft-forming optimum F2 gel encapsulated the EMP-copolymeric nanoparticles after 8 h of exposure to the 0.1 N HCl.

Where i = dissolution sample numbers, n = number of dissolution times, t = time at the midpoint between times ti and ti-1, ΔM = amount of drug dissolved between times ti and ti-1. MDT is the time required for 50 % drug release. A higher mean dissolution time value suggests a slower release rate [20]. As shown in Table 5, F2 had the lowest IDR of burst release and highest MDT_6.8_ values compared to the F1 and F3 formulations, which could contribute to PEG and SA hydrophilic interactions and hydrogen bond formation for raft formation of the optimum SA, PEG, and EMP combinations in contact with 0.1 N HCl. In addition, Fig. 4B shows a similarity between the F1 and F3 formulations' release behavior (Similarity Factor [20] = 76.02 ± 0.00 “≥60”). The F2 formulation's release profile (Fig. 4B) was between the poorly soluble raw EMP drug and the EMP-SA-PEG-NPs. Moreover, the F2 formulation's release results indicated a higher release rate (MDT_1.2_) in the simulated gastric media compared to the F1 and F3 formulations, and the results showed a lower release rate (MDT_6.8_) in the simulated intestinal media compared to the F1 and F3 formulation. The overall release profile of the F2 formulation was compatible with having a controlled release formula with minimal burst release, a higher rate of dissolution in the simulated gastric media compared to the simulated intestinal media, and the ability to release the EMP-nanoparticles with better dissolution compared to the free drug at the gastric region as the action site. All these parameters were supported by the ability of the formulation to float instantaneously when in contact with the simulated gastric media. To support that, the raft characterizations of F1, F2 and F3 formulations are listed in Table 6. The raft characterizations of the F2 formulation showed the most durable, strongest, and lowest density raft compared to the F1 and F3 formulations. The F2 raft formed after 8 h of exposure to 0.1 N HCl as illustrated in Fig. 5. F2 formulation release and raft characterizations findings were correlated to the strong interaction between SA, PEG and EMP induced after exposure to the simulated gastric media [22,37]. In Fig. 5, the insoluble floating gel “raft” was formed in the presence of the simulated gastric media (0.1 N HCl). This is related to alginate gel conversion to alginic acid in an acidic media [20].

**Table 5:** Parameters of EMP release in 0.1 N HCl and media of pH 6.8 from different raft-forming gels of EMP-ALG-PEG-NPs. *Each value is the average of n=3 ± SD*.

Formulation	IDR (mg/min)	MDT_1.2_^a^ (hr)	MDT_6.8_^b^ (hr)
**F1**	0.54 ± 0.04	17.64 ± 0.02	16.80 ± 0.02
**F2**	0.49 ± 0.02	13.98 ± 0.04	17.54 ± 0.03
**F3**	0.57 ± 0.05	14.05 ± 0.03	15.64 ± 0.05

a MDT_1.2_ = Mean dissolution time for the first 2 h in 0.1 N HCl.

b MDT_6.8_ = Mean dissolution time for the last 4 h in pH 6.8.

**Table 6:** Raft characterizations of different products.

#	Raft weight (g) ±SD^a^	Raft volume (mL) ±SD^a^	Raft strength (g) ± SD^a^	Raft durability (min)
				Median
F1	1.50 ± 0.12	10.21 ± 0.33	10.30 ± 0.33	80
F2	1.22 ± 0.30	9.11 ± 0.53	20.11 ± 0.12	≥120
F3	1.44 ± 0.21	9.22 ± 0.31	16.00 ± 0.22	90

**Abbreviation**	**Full Name**
EMP	Empagliflozin
SA	Alginate
PEG	Polyethelene glycol
NPs	Nanoparticles
EMP-ALG-PEG-NPs	EMP-loaded Alginate-Ploy Ethylene Glycol nanoparticles
EMP co-polymeric	EMP-loaded Alginate-Ploy Ethylene Glycol nanoparticles
ALG-PEG-NPs	Alginate-Ploy Ethylene Glycol nanoparticles
Blank co-polymeric NPs	Alginate-Ploy Ethylene Glycol nanoparticles
MDT	Medium Dissolution Time
IDR	Initial Dissolution Rate

a For n = 3 ± SD.

### 3.5 Anti-cancer actions of the EMP nanoparticles

**Fig. 6:**
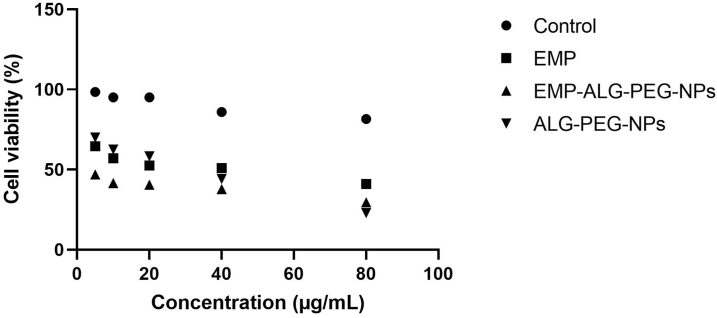
The effect of MTT assay in the different treatment groups in comparison to the control. *One-way ANOVA was to determine the significance between the EMP-ALG-PEG-NPs with EMP (@), ALG-PEG-NPs ($), and control (#), for which the P-value of less than 0.05 was considered significant.*

MTT assay is an extensively used colorimetric assay for estimating metabolic activities. In normal physiological conditions, NAD(P)H-dependent oxidoreductase present in the cytosol of the cell provides a concrete idea about the viable number of cells. Interestingly, enzymes react with the MTT assay's tetrazolium dye and convert it into insoluble formazan (purple color). This colour change is only possible when the cell is viable and the mitochondrial reductase enzyme is active. Thus, based on the absorbance captured at 570 nm, cell viability or IC_50_ can be calculated. Hence, IC_50_ or this process (MTT assay) provides important information about the anti-cancer potential of an investigational drug [15]. As a result, in this investigation, we used the MTT test to assess the anti-cancer potential of our optimized produced EMP-ALG-PEG-NPs and compared them to ALG-PEG-NPs and EMP. The study found that EMP-ALG-PEG-NPs had the lowest IC50 value (6.02 ± 0.29 μg/mL), followed by EMP (29.91 ± 0.95 μg/mL) and ALG-PEG-NPs (78.62 ± 5.37 μg/mL) (Fig. 6). Moreover, based on the MTT assay, it was inferred that EMP-ALG-PEG-NPs exceeded by three times the EMP potency and exceeded by ten times the ALG-PEG-NPs potency against stomach cancer cells. The findings were correlated to the dissolution enhancement, better cellular entrapment, and nanoparticles that boosted the sensitivity of the EMP-ALG-PEG-NPs compared to the other groups [10].

### 3.6 Anti-inflammatory effects of EMP nanoparticles

**Fig. 7:**
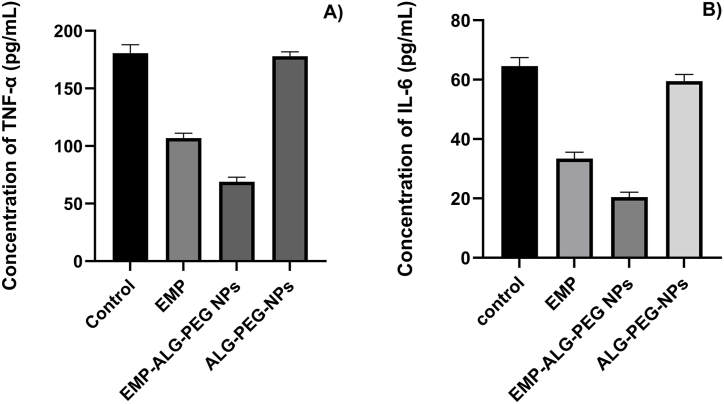
Showing the anti-inflammatory effects of various groups on the level of (A) TNF-α and (B) IL-6. One-way ANOVA was to determine the significance between the EMP-ALG-PEG-NPs with EMP (*), ALG-PEG-NPs ($), and control (#), for which the P-value of less than 0.05 was considered significant.

Blocking or reducing the expression of NF-κB and other pro-inflammatory cytokines appears to be an important therapeutic modality in the treatment and management of stomach cancer [1]. Hence, in the present study, we checked the effect of various developed formulations and EMP for their anti-inflammatory effect. The outcomes of the study showed a significant increase in the level of TNF-α (Fig. 7A) and IL-6 (Fig. 7B) in the control group compared to EMP-ALG-PEG-NPs by 2.57 and 3.19 fold, respectively (p < 0.001). Moreover, when the anti-inflammatory effect was compared between EMP-ALG-PEG-NPs and ALG-PEG-NPs and between EMP-ALG-PEG-NPs and EMP, it was found that EMP-ALG-PEG-NPs significantly reduced the level of TNF-α and IL-6 as compared to ALG-PEG-NPs (p < 0.001 for TNF-α and IL-6 by 2.45 and 2.94 folds, respectively), and EMP (p < 0.001 for TNF-α and p < 0.01 for IL-6, by 1.48 and 1.72 folds, respectively) (Fig. 7). The findings were correlated to the dissolution enhancement, better cellular entrapment, and nanoparticles boosted sensitivity of the EMP-ALG-PEG-NPs compared to the other groups [10]. Thus, based on the outcome of the inflammatory study, the results justify the rationale for repurposing the EMP-ALG-PEG-NPs for the treatment and management of stomach cancer and associated gastritis.

## 4 Conclusion

EMP is known for its poor safety and efficacy profile due to its fast body distribution and poor solubility. An oral long-acting and floating/raft-forming nano gel was optimized to release coated EMP nanoparticles, and the released EMP nanoparticles showed enhanced dissolution compared to raw EMP particles. In addition, the EMP-ALG-PEG-NPs after release from the optimized raft were designed to have an enhanced dissolution profile compared to the EMP raw materials. The solubility of EMP nanoparticles was enhanced in 0.1 N HCl and pH 6.8 by 4.96 ± 0.03 and 11.33 ± 0.02 fold, respectively, compared to the raw EMP powder. This study developed a novel EMP-ALG-PEG-NPs using SA and PEG, which showed distinctive interactions, morphology, and crystallinity compared to free EMP. As a result, these unique properties were the foundation of enhanced EMP-ALG-PEG-NPs dissolution in the simulated gastrointestinal tract media over time. The success and rationale of developing optimized NPs against stomach cancer were studied and validated, where a significant and superior anti-cancer effect was achieved after treatment with EMP-ALG-PEG-NPs. Based on the outcome of the inflammatory study, the rationale for repurposing the EMP-ALG-PEG-NPs for the treatment and management of stomach cancer and associated gastritis were justified. Moreover, the EMP-ALG-PEG-NPs are suggested for oral administration to enhance EMP efficacy and intestinal absorption for anti-cancer treatment. Future studies can investigate the formulation gel stability and EMP absorption using *in vivo* models to ensure the resilience and application of the *in situ* forming gel.

## Funding

10.13039/501100021772The Deanship of Scientific Research (DSR) at 10.13039/501100004054King Abdulaziz University (KAU), Jeddah, Saudi Arabia has funded this project, under grant no. (RG-21–166–43).

## Data availability statement

All data is listed in the manuscript. The supplementary and background trials and data are available on request.

## CRediT authorship contribution statement

**Nabil A. Alhakamy:** Writing – review & editing, Supervision, Investigation, Funding acquisition, Formal analysis, Data curation. **Samaa Abdullah:** Writing – original draft, Visualization, Validation, Supervision, Software, Project administration, Methodology, Investigation, Formal analysis, Data curation, Conceptualization. **Shadab Md:** Writing – review & editing, Methodology, Funding acquisition, Data curation. **Akhalakur Rahman Ansari:** Writing – review & editing, Resources, Project administration. **Subrat Kumar Bhattamisra:** Writing – review & editing, Software, Resources, Data curation. **Ibrahim M. Ibrahim:** Supervision, Software, Funding acquisition, Data curation. **Hadil Alahdal:** Funding acquisition. **Abeer A. Altamimi:** Funding acquisition. **Rasheed A. Shaik:** Project administration, Methodology, Funding acquisition.

## Declaration of competing interest

The authors declare that they have no known competing financial interests or personal relationships that could have appeared to influence the work reported in this paper.

## References

[1] Kamal G., Abdullah S., Basingab F., Bani-Jaber A., Hamdan I. (2022). Curcumin-betaine solid dispersion for enhancing curcumin dissolution and potentiating pharmacological synergism in gastric cancer cells. J. Drug Deliv. Sci. Technol..

[2] Zhou H., Wang S., Zhu P., Hu S., Chen Y., Ren J. (2018). Empagliflozin rescues diabetic myocardial microvascular injury via AMPK-mediated inhibition of mitochondrial fission. Redox Biol..

[3] Abdelhamid A.M., Saber S., Youssef M.E., Gaafar A.G.A., Eissa H., Abd-Eldayem M.A., Alqarni M., Batiha G.E.-S., Obaidullah A.J., Shahien M.A., El-Ahwany E., Amin N.A., Etman M.A., Kaddah M.M.Y., Abd El-Fattah E.E. (2022). Empagliflozin adjunct with metformin for the inhibition of hepatocellular carcinoma progression: emerging approach for new application. Biomed. Pharmacother..

[4] Scheen A.J. (2014). Pharmacokinetic and pharmacodynamic profile of empagliflozin, a sodium glucose co-transporter 2 inhibitor. Clin. Pharmacokinet..

[5] He L., Shang Z., Liu H., Yuan Z.-x. (2020). Alginate-based platforms for cancer-targeted drug delivery. BioMed Res. Int..

[6] Caccamo M.T., Magazù S. (2017). Ethylene glycol - polyethylene glycol (EG-PEG) mixtures: infrared spectra wavelet cross-correlation analysis. Appl. Spectrosc..

[7] Manoel J.W., Primieri G.B., Volpato N.M., Steppe M. (2021). Development and validation of a dissolution test for empagliflozin in film-coated tablets. Drug Analytical Research.

[8] Stefansson M. (1999). Assessment of alginic acid molecular weight and chemical composition through capillary electrophoresis. Anal. Chem..

[9] Abdullah S., El Hadad S., Aldahlawi A. (2021). In vitro optimization, characterization and antitumor evaluation against colorectal cancer of a novel 5Fluorouracil fluorouracil oral nanosuspension using soy protein, polysaccharides-protein complexation, and in-situ gel formation. J. Drug Deliv. Sci. Technol..

[10] Abdullah S., El Hadad S., Aldahlawi A. (2022). The development of a novel oral 5-Fluorouracil in-situ gelling nanosuspension to potentiate the anticancer activity against colorectal cancer cells. Int. J. Pharm..

[11] Md S., Abdullah S., Alhakamy N.A., Alharbi W.S., Ahmad J., Shaik R.A., Ansari M.J., Ibrahim I.M., Ali J. (2021). Development, optimization, and in vitro evaluation of novel oral long-acting resveratrol nanocomposite in-situ gelling film in the treatment of colorectal cancer. Gels.

[12] Md S., Abdullah S., Alhakamy N.A., Shaik R.A., Ansari A.R., Riadi Y., Ahmad J., Ali R., Gorain B., Karim S. (2022). Sustained-release ginseng/sodium alginate nano hydrogel formulation, characterization, and in vivo assessment to facilitate wound healing. J. Drug Deliv. Sci. Technol..

[13] Md S., Abdullah S., Alhakamy N.A., Shaik R.A., Eldakhakhny B.M., Omar U.M., Eid B.G., Ansari A.R., Alamoudi A.J., Rizg W.Y., Riadi Y., Venkateswaran S.P., Rashid M.A. (2022). Development and evaluation of ginkgo biloba/sodium alginate nanocomplex gel as a long-acting formulation for wound healing.

[14] Md S., Abdullah S., Awan Z., Alhakamy N.A. (2022). Smart oral pH-responsive dual layer nano-hydrogel for dissolution enhancement and targeted delivery of naringenin using protein-polysaccharides complexation against colorectal cancer. J. Pharmaceut. Sci..

[15] Md S., Abdullah S.T., Alhakamy N.A., Bani-Jaber A., Radhakrishnan A.K., Karim S., Shahzad N., Gabr G.A., Alamoudi A.J., Rizg W.Y. (2021). Ambroxol hydrochloride loaded gastro-retentive nanosuspension gels potentiate anticancer activity in lung cancer (A549) cells. Gels.

[16] Gavas S., Quazi S., Karpiński T.M. (2021). Nanoparticles for cancer therapy: current progress and challenges. Nanoscale Res. Lett..

[17] Gamboa A., Araujo V., Caro N., Gotteland M., Abugoch L., Tapia C. (2015). Spray freeze-drying as an alternative to the ionic gelation method to produce chitosan and alginate nano-particles targeted to the colon.

[18] Md S., Alhakamy N.A., Aldawsari H.M., Husain M., Kotta S., Abdullah S.T., A. Fahmy U., Alfaleh M.A., Asfour H.Z. (2020). Formulation design, statistical optimization, and in vitro evaluation of a naringenin nanoemulsion to enhance apoptotic activity in A549 lung cancer cells. Pharmaceuticals.

[19] Li Q., Cao Q., Yuan Z., Wang M., Chen P., Wu X. (2022). A novel self-nanomicellizing system of empagliflozin for oral treatment of acute pancreatitis: an experimental study. Nanomed. Nanotechnol. Biol. Med..

[20] Bani-Jaber A., Abdullah S. (2020). Development and characterization of novel ambroxol sustained-release oral suspensions based on drug-polymeric complexation and polymeric raft formation. Pharmaceut. Dev. Technol..

[21] Dettmar P.W., Gil-Gonzalez D., Fisher J., Flint L., Rainforth D., Moreno-Herrera A., Potts M. (2018). A comparative study on the raft chemical properties of various alginate antacid raft-forming products. Drug Dev. Ind. Pharm..

[22] Kapadia C.J., Mane V.B. (2007). Raft-forming agents: antireflux formulations. Drug Dev. Ind. Pharm..

[23] Frampton J.E. (2018). Empagliflozin: a review in type 2 diabetes. Drugs.

[24] Hampson F.C., Farndale A., Strugala V., Sykes J., Jolliffe I.G., Dettmar P.W. (2005). Alginate rafts and their characterisation. Int. J. Pharm..

[25] Hampson F.C., Jolliffe I.G., Bakhtyari A., Taylor G., Sykes J., Johnstone L.M., Dettmar P.W. (2010). Alginate–antacid combinations: raft formation and gastric retention studies. Drug Dev. Ind. Pharm..

[26] Eliaa S.G., Al-Karmalawy A.A., Saleh R.M., Elshal M.F. (2020). Empagliflozin and doxorubicin synergistically inhibit the survival of triple-negative breast cancer cells via interfering with the mTOR pathway and inhibition of calmodulin: in vitro and molecular docking studies. ACS Pharmacol. Transl. Sci..

[27] Xie Z., Wang F., Lin L., Duan S., Liu X., Li X., Li T., Xue M., Cheng Y., Ren H., Zhu Y. (2020). An SGLT2 inhibitor modulates SHH expression by activating AMPK to inhibit the migration and induce the apoptosis of cervical carcinoma cells. Cancer Lett..

[28] Douglas S.J., Davis S.S., Illum L. (1987). Nanoparticles in drug delivery. Crit. Rev. Ther. Drug Carrier Syst..

[29] Burin S.L., Lourenço R.L., Doneda M., Müller E.I., Paula F.R., Adams A.I.H. (2021). Development of an HPLC-UV method to assay empagliflozin tablets and identification of the major photoproduct by quadrupole time-of-flight mass spectrometry. J. Chromatogr. Sci..

[30] Niguram P., Polaka S.N., Rathod R., Kalia K., Kate A.S. (2020). Update on compatibility assessment of empagliflozin with the selected pharmaceutical excipients employed in solid dosage forms by thermal, spectroscopic and chromatographic techniques. Drug Dev. Ind. Pharm..

[31] Mourdikoudis S., Pallares R.M., Thanh N.T.K. (2018). Characterization techniques for nanoparticles: comparison and complementarity upon studying nanoparticle properties. Nanoscale.

[32] Biswal S., Sahoo J., Murthy P.N., Giradkar R.P., Avari J.G. (2008). Enhancement of dissolution rate of gliclazide using solid dispersions with polyethylene glycol 6000. AAPS PharmSciTech.

[33] Biswal S., Sahoo J., Murthy P.N., Giradkar R.P., Avari J.G. (2008). Enhancement of dissolution rate of gliclazide using solid dispersions with polyethylene glycol 6000. AAPS PharmSciTech.

[34] El-Ashmawy A.A., Abdelfattah F.M., Emara L.H. (2022). Novel glyceryl monostearate- and polyethylene glycol 6000-based ibuprofen pellets prepared by hot-melt extrusion: evaluation and stability assessment. J. Pharmaceut. Innov..

[35] Le Pevelen D.D., Lindon J.C. (2010). Encyclopedia of Spectroscopy and Spectrometry.

[36] Bani-Jaber A., Alshawabkeh I., Abdullah S., Hamdan I., Ardakani A., Habash M. (2017). In vitro and in vivo evaluation of casein as a drug carrier for enzymatically triggered dissolution enhancement from solid dispersions. AAPS PharmSciTech.

[37] Giunchedi P., Gavini E., Moretti M.D.L., Pirisino G. (2000). Evaluation of alginate compressed matrices as prolonged drug delivery systems. AAPS PharmSciTech.

